# Genetic variation within the gene encoding the HIV-1 CCR5 coreceptor in two South African populations

**DOI:** 10.1016/j.meegid.2010.02.012

**Published:** 2010-05

**Authors:** Anabela C.P. Picton, Maria Paximadis, Caroline T. Tiemessen

**Affiliations:** a AIDS Virus Research Unit, National Institute for Communicable Diseases, Johannesburg, South Africa; b Department of Virology, University of the Witwatersrand, Johannesburg, South Africa

**Keywords:** CCR5, HIV-1, South African population, Genetic variation

## Abstract

Polymorphisms within the open reading frame as well as the promoter and regulatory regions can influence the amount of CCR5 expressed on the cell surface and hence an individual's susceptibility to HIV-1. In this study we characterize *CCR5* genes within the South African African (SAA) and Caucasian (SAC) populations by sequencing a 9.2 kb continuous region encompassing the *CCR5* open reading frame (ORF), its two promoters and the 3′ untranslated region. Full length *CCR5* sequences were obtained for 70 individuals (35 SAA and 35 SAC) and sequences were analyzed for the presence of single-nucleotide polymorphisms (SNPs), indels and intragenic haplotypes. A novel SNP (+258G/C) within the ORF leading to a non-synonomous amino acid (Trp → Cys) change was detected in one Caucasian individual. Results demonstrate a high degree of genetic variation: 68 SNP positions, four indels, as well as the Δ32 deletion mutant, were detected. Seven complex putative haplotypes spanning the length of the sequenced region have been identified. These haplotypes appear to be extensions of haplotypes previously described within *CCR5*. Two haplotypes, SAA-HHE and SAC-HHE were found in high frequency in the SAA and SAC population groups studied (20.0% and 18.6%, respectively) and share four SNP positions suggesting an evolutionary link between the two haplotypes. Only one of the identified haplotypes, SAA/C–HHC, is common to both study populations but the haplotype frequency differs markedly between the two groups (8.6% in SAA and 52.9% in SAC). The two population groups show differences in both haplotype arrangement as well as SNP profile.

## 1 Introduction

The human coreceptor, CCR5, acts as the principal coreceptor required for macrophage-tropic (R5) human immunodeficiency virus type 1 (HIV-1) virions to gain entry to a cell (Deng et al., 1996; Dragic et al., 1996). Shortly after the role of CCR5 was discovered, the *CCR5*Δ*32* mutant and its association with protection to HIV-1 infection in individuals homozygous for this allele, was found (Samson et al., 1996). This discovery provided the first genetic evidence of protection to HIV-1 infection and prompted further studies of the gene and how its naturally occurring mutations may influence the outcome of HIV-1 exposure and infection.

The *CCR5* gene is composed of four exons and two introns, where exons 2A and 2B are not interrupted by an intron (Mummidi et al., 1997). Exon 3 contains an intronless open reading frame (ORF). Two *CCR5* promoters have been described, a weak upstream promoter (P_U_ or P2) and a stronger downstream promoter (P_D_ or P1) (Mummidi et al., 1997). Cell surface expression of CCR5 is highly variable, even in individuals homozygous for the wild type ORF region. This may be explained by differences in the promoter region of *CCR5* which result in differing CCR5 expression levels (Menten et al., 2002). To date, several mutations and single-nucleotide polymorphisms (SNPs) in the HIV-1 coreceptor gene, *CCR5*, have been found to be important genetic factors capable of influencing susceptibility to HIV-1 infection or affecting the rate of disease progression.

Striking ethnic or population differences in SNP frequencies of *CCR5* exist. The most studied polymorphism exhibiting this is the *CCR5*Δ*32* mutant. The *CCR5*Δ*32* allele occurs at a variable frequency of 4–15% in Caucasian populations, with an average of 10% in Europe (reviewed in Galvani and Novembre, 2005) and yet is rarely found in Asian or African populations. In a South African context, Petersen et al. (2001) identified seven novel mutations within the *CCR5* ORF in African and Coloured populations, however polymorphisms within the promoter region have not been studied. Thus, a detailed descriptive study of SNPs and/or haplotypes within the *CCR5* receptor gene was carried out in two South African populations, South African Africans (SAA) and South African Caucasians (SAC), with the aim of providing a baseline study of the prevalence of polymorphisms that exist in *CCR5* within these populations and to determine whether all previously defined haplotypes are represented within these populations.

## 2 Materials and methods

### 2.1 Study population

Characterization of the *CCR5* gene was carried out on 70 healthy, HIV-1 uninfected adult volunteers, 35 were SAA and 35 were SAC. This study was approved by the University of the Witwatersrand Committee for Research on Human Subjects, and informed written consent was obtained from all participants.

### 2.2 PCR and sequencing of CCR5

Genomic DNA was extracted from blood samples anticoagulated with ethylenediaminetetraacetic acid (EDTA) using QIAamp DNA Mini Kit (QIAGEN, Dusseldorf, Germany). A ∼9.2 kb continuous region encompassing the *CCR5* open reading frame (ORF), its two promoters and the 3′ untranslated region (UTR) was polymerase chain reaction (PCR) amplified in five overlapping sections using Expand High Fidelity PCR System (Roche, Mannheim, Germany). PCR and sequencing primers were designed using PRIMER DESIGNER for Windows (v. 2.0) (Supplementary Table S1) using the published sequences for *CCR5* (GenBank accession: U95626, AF017632 (Moriuchi et al., 1997), AF031236 and AF031237 (Mummidi et al., 1997)) as reference sequences.

All sequencing reactions were carried out using BigDye Terminator version 3.1 chemistry (Applied Biosystems, Foster City, CA, USA). Amplified fragments were sequenced using the automated 3100 Genetic Analyzer (Applied Biosystems).

### 2.3 Sequence analysis

Sequence data was assembled and analyzed for the presence of SNPs and indels using SEQUENCHER software version 4.5 (Gene Codes Corporation, Ann Arbor, MI, USA). Assembled sequences were aligned with each other and the published GenBank sequence, U95626, using SEQUENCHER, to identify polymorphisms. The GenBank NCBI SNP database (dbSNP) was searched for all reported SNPs in the *CCR5* gene to determine whether polymorphisms detected in this study had been previously reported.

The *CCR5* numbering system used in this study is as described by Mummidi et al. (2000) where the first nucleotide of the translational start site is designated as +1 and the nucleotide immediately upstream from that is −1. A composite of the reference sequences AF031236 and AF031237 (Mummidi et al., 1997) was used as a basis for determining SNP positions as these sequences appeared to be closer to the wild type (WT) or more ‘ancestral’ gene. It must be noted however that when all *Homo sapiens* reference sequences used in this study were aligned, a number of differences between them were noted, including base insertions or deletions (indels), which would affect the SNP position values. Also, AF031236 and AF031237 do not encompass the entire region sequenced in our study. Thus, using the sequences flanking the various SNPs may be a more reliable means of identifying SNP positions (Supplementary Table S2).

### 2.4 Determination of wild type (WT) reference sequence

Once polymorphisms within *CCR5* were identified, it was necessary to determine which nucleotide to deem as the WT nucleotide. Generally, the most prevalent nucleotide in our combined populations was considered to be the WT or ancestral nucleotide/allele. In addition, to identify the WT nucleotide where it was not apparent which nucleotide/allele was most prevalent, the human *CCR5* sequences were aligned with those of the chimpanzee found on the sequence available for *Pan troglodytes* chromosome 3 (GenBank accession number: NW_001232822.1) and the *Pan troglodytes CCR5* sequence (GenBank accession numbers: NM_001009046 and AF005663).

### 2.5 Inference of putative haplotypes

Analysis of the sequence data generated for *CCR5* revealed certain obvious patterns wherein the presence of a polymorphism at one position was consistently associated with polymorphisms at one or more other positions. These associations were identified as putative intragene haplotypes. The HAPLOTYPER software which uses a Bayesian algorithm for haplotypes inference (Niu et al., 2002) was also used to infer haplotypes for *CCR5*.

The frequencies of putative intragenic haplotypes were calculated by counting the number of alleles harbouring the haplotypes and dividing by the total number of alleles. Counting of the haplotypes was irrespective of the presence of additional SNPs not forming part of the haplotypes in question.

### 2.6 Characterization of indels

Five SAA and one SAC individual appeared to have a previously unidentified indel downstream from the open reading frame (+2772). Characterization of the putative indel as well as verification of Intron 2 indels was carried out by TA cloning of PCR amplicons into the pCR^®^4-TOPO^®^ cloning vector using the TOPO-TA Cloning Kit (Invitrogen, Carlsbad, CA, USA). Recombinant plasmids were screened for the allele with the putative indel by sequencing. Sequences were aligned with reference sequences, using SEQUENCHER, in order to characterize the indel.

### 2.7 Hardy–Weinberg equilibrium

All polymorphic loci detected within the characterized *CCR5* gene region were tested for deviation from Hardy–Weinberg equilibrium using the conventional Monte Carlo exact test of Guo and Thompson (1992) implemented through the computer program TFPGA (Miller, 1997). The two population groups were tested independently.

### 2.8 Linkage disequilibrium between haplotype SNPs

To test whether the SNPs forming part of the putative intragenic haplotypes were in complete or strong linkage, linkage disequilibrium between every two SNP combination in each haplotype was estimated using the method described by Lewontin (1964) where the linkage disequilibrium coefficient *D* was calculated (D_*ij*_ = HF_*ij*_ − *p*_*i*_*p*_*j*_). *D* was subsequently normalized (*D*′) or standardized by the maximum value it can take (*D*_max_) using the formula *D*′_*ij*_ = *D*_*ij*_/*D*_max_ where HF_*ij*_ is the frequency of the haplotypes carrying SNPs *i* and *j*, *p*_*i*_ and *p*_*j*_ are the frequencies of SNPs *i* and *j*, respectively and *D*_max_ is either min [p_*i*_p_*j*_, (1 − *p*_*i*_)(1 − *p*_*j*_) if *D*_*ij*_ < 0, or min [(1 − *p*_*i*_) *p*_*j*_, *p*_*i*_(1 − *p*_*j*_)] if *D*_*ij*_ > 0. *D*′ values are defined in the range [−1, 1] with a value of ‘1’ representing perfect disequilibrium. The statistical significance of the linkage disequilibrium between each of the SNP pairs was evaluated by the approximate chi-square described by Liau et al. (1984).

### 2.9 Fisher exact test

Fisher exact tests were performed using the Simple Interactive Statistical Analysis software (Uitenbroek, D. G., Binomial. SISA. 1997. http://www.quantitativeskills.com/sisa/distributions/binomial.htm. [1 January 2004]) to test whether there was any significant difference in SNP frequencies between this and other studies.

## 3 Results

### 3.1 Single-nucleotide polymorphisms

**Fig. 1:**
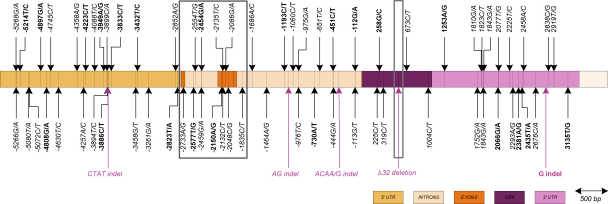
Schematic representation of *CCR5* gene region sequences indicating polymorphism positions and nucleotide base changes at these points. The structure of the gene (Mummidi et al., 1997) is indicated by colour coded boxes. Nucleotide base changes at SNP positions are described by stating the WT base first followed by the base found on the minor allele. Grey boxes delineate regions of the *CCR5* gene which have been previously used to describe *CCR5* haplotypes. Newly identified (NI) polymorphisms and indels are indicated in bold font. UTR: untranslated region, ORF: open reading frame.

Assembled sequences of the *CCR5* gene including promoter, coding and 3′ UTR regions, from 70 HIV-1 uninfected individuals were analyzed for DNA polymorphisms, SNPs and indels. Across the entire 9.2 kb region sequenced, 68 SNPs were identified. The positions and nucleotide (nt) changes are indicated in Fig. 1. The identified polymorphisms were found across the entire sequenced region with the exclusion of exon 2B, a small region spanning 54 nucleotides. The majority of the polymorphisms were located in the intron and UTR of the gene and only six were located in the ORF (Fig. 1). With regards to the two study populations, 60 and 37 polymorphisms were found in the SAA and SAC populations, respectively. Of the 68 identified SNPs, 46 have been previously described in the GenBank dbSNP database and by Petersen et al. (2001). Their corresponding accession numbers, where available, are shown in Table 1. To the best of our knowledge, with comparison to the GenBank dbSNP database and literature reports, 24 polymorphisms are newly identified and have been designated as newly identified (NI) in Table 1. These NI polymorphisms were found in both population groups. Newly identified polymorphisms are also distributed across the entire gene although the majority are located in the 5′ and 3′ UTRs. Most NI SNPs were found to be rare polymorphisms present in only one individual. Exceptions to this were the NI polymorphisms, −4223C/T, −3886C/T, −2454G/A and −451C/T, which were detected in higher numbers (three or four individuals each, all of which were heterozygous for the polymorphisms) in either/both populations.

The alignment of reference sequences (GenBank accession numbers: U95626, AF031236, AF031237, AF017632 and NT_022517.17) did not demonstrate 100% homology at many of the SNP positions detected, as well as at other potential polymorphic positions not detected in this study. Also, it was not always apparent which the most predominant base at certain positions was. For instance, with the −2554G/T SNP, 79 (56.4%) and 61 (43.6%) alleles in this study contained a G and T nucleotide, respectively. Although the G allele was more frequent overall, in the SAC population the major nucleotide was a T (55.7%) and in SAA individuals the major alleles was a G (68.6%). Caution was necessary in the selection of a WT nucleotide as the population sizes used in this study were of a size where bias could be introduced and the apparent WT allele (most frequent) may not correspond to the ancestral allele. Thus, reference *Homo sapiens* sequences were aligned with *Pan troglodytes* sequences. Due to the low mutation rate since the human–chimpanzee divergence, the human allele almost always corresponds to the allele present in chimpanzees (Cargill et al., 1999). Where an allele which was obviously the major allele in *Homo sapiens* (this study and sequences used as references) was not in agreement with that of the *Pan troglodytes* sequences at the same position, the former was selected as WT. This was the case for the indels, CTAT/–, AG/– and ACAA/G where published sequences for *Pan troglodytes*, some of the *Homo sapiens* reference sequences, as well as data for SAC would indicate the minor allele contained nucleotide insertions at these indel positions, yet overall the most frequent alleles detected in this study indicated that the minor allele for all 3 indel positions was the allele containing nucleotide deletions at those positions. The same was observed for SNPs −2852A/G and −113G/T, where the *Pan troglodytes* base at that position was the equivalent of the minor allele/nucleotide in humans. The minor allele frequencies of the *CCR5* SNPs and indels in both the study populations are shown in Table 1.

Of the polymorphisms detected within the *CCR5* ORF, one has not been reported previously (Table 1). This novel SNP (+258G/C) within the ORF leads to a non-synonymous amino acid change (Trp → Cys) at codon 86 and was detected in one SAC individual heterozygous for that mutation. Four mutations in the open reading frame were detected in the SAA population: +225T/C; +319C/T; +673C/T and +1004C/T (S75S; L107F; R225X and A335 V, respectively). One individual was found to be heterozygous for previously described mutations at both codon 107 and 225 (Petersen et al., 2001). In a study conducted by Petersen et al. (2001), these two mutations were reported as occurring simultaneously in an individual and at low frequencies in SAA and South African Coloureds. The codon 335 amino acid substitution mutation was detected within the SAA population at a frequency of 0.071 (*n* = 5) and only one individual in the SAC population was found to harbour this mutation (Table 1). Previous reports looking at African American (Ansari-Lari et al., 1997; Carrington et al., 1997; Carrington et al., 1999) and SAA (Petersen et al., 2001) populations found the mutation present at a frequency of approximately 3% and 2%, respectively. Although representation of this SNP appears higher (7.1%) in our study, this did not differ statistically from frequencies reported in these studies (*P* > 0.05).

Several polymorphisms were found to be restricted to either the SAA or the SAC population group (frequencies highlighted in grey in Table 1). The SNPs, −3894T/C, −3261G/A, −2132C/T, −1686A/C, −1464A/G, −113G/T and +1752G/A, are all restricted to the SAA population at a frequency of >18% and all form part of the putative haplotypes, SAA-HHA and SAA-HHD, identified in this study. In addition, the SNPs, −4745C/T, +1843G/A and +1846G/A, were detected at a reasonably high frequency of 12.9% in SAA individuals but not in SAC individuals. There were no SNPs found exclusively in the SAC population which were also present at relatively high frequencies (i.e., at a frequency >10%) in that population. Where the SNP frequency is low in one population, absence in the other population cannot be used to state that that particular SNP is only prevalent in one population due to the sample size used in this study.

### 3.2 Indels

Five indels were detected across the entire *CCR5* gene (Fig. 1). Four of the five detected indels have been previously described (Dean et al., 1996; Samson et al., 1996; Mummidi et al., 1997). The Δ32 deletion indel was found in 5/35 (14.3%) SAC individuals, all of which were heterozygous for this mutation, but not in SAA individuals. In a previous South African study, the *CCR5*Δ*32* allele was detected at a frequency of 9.4% and 0.1% in SAC and SAA individuals, respectively (Williamson et al., 2000). Comparison of *CCR5*Δ*32* allele frequencies observed in SAC populations in the two studies showed no significant difference between them (*P* = 0.68). The other three previously reported deletion indels (CTAT/–, AG/– and ACAA/G) appear to be in very strong linkage disequilibrium (*D*′ = 1.0, *P* < 0.0005 for all three indel associations in both population groups).

Two indels are located within Intron 2 of the *CCR5* gene. The indel located at position −362 has been reported differently. In a report characterizing the *CCR5* gene this is shown to be a CAA indel (Mummidi et al., 1997). Within the dbSNP database there are two polymorphism reports for that location: accession number rs41515644 reports an A/G SNP at that position, whereas accession number rs71615644 reports that the four nucleotide sequence, ACAA, is substituted with a single guanine nucleotide. Within our study group, only the latter polymorphism was observed. The Intron 2 PCR amplicons from two individuals heterozygous for the ACAA indel were cloned and sequenced. Sequencing demonstrated that on the alleles where there was a ACAA deletion, there would be a G substitution at that point. Thus, in our report, the deletion and base substitution have been treated as a single polymorphism. It is possible though that the ACAA/G indel may have arisen as two separate events which became evolutionarily linked, i.e. an A to G substitution and a CAA deletion immediately downstream from the substitution.

The indel downstream from the open reading frame (Fig. 1) consists of a single guanine insertion. Alleles containing the indel have a string of nine guanine bases in that region, whereas the WT alleles have eight guanine nucleotides. The exact position of the single base insertion within the eight consecutive guanine bases of the WT sequence cannot be precisely determined. Thus, the position of +2772, at the end of the eight guanine bases has been selected.

### 3.3 Haplotypes

**Fig. 2:**
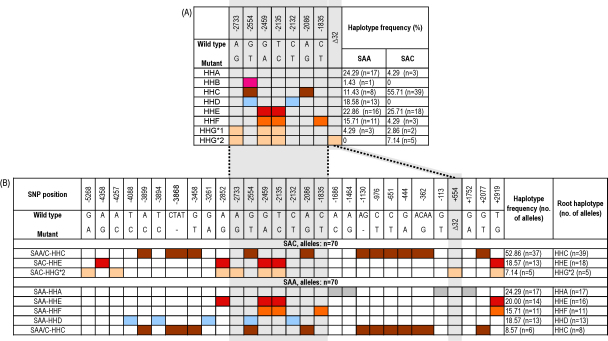
(A) Schematic representation of haplotypes previously defined by Gonzalez et al. (1999) and the frequency at which they were detected within our two study populations. (B) Haplotypes identified within the *CCR5* gene in the SAA and SAC study populations. Haplotypes were named by prefixing the root haplotype with the population within which it was found. Polymorphic positions as well as the wild type (WT) to mutant change are shown. Coloured boxes indicate SNPs or indels which form part of the haplotype and the frequency of occurrence in that particular population group is indicated. The root haplotype (Gonzalez et al., 1999) is also indicated.

Individuals within the SAA and SAC populations were assigned to previously described haplogroups (Gonzalez et al., 1999) based on SNPs at positions −2733, −2554, −2459, −2135, −2132, −2086 and −1835 as well as the presence of *CCR5*Δ*32* (Fig. 2A). One SAA individual was found to be heterozygous for a haplotype allele which could not be classed into any of the haplotypes defined by Martin et al. (1998) or Gonzalez et al. (1999). Similar trends in haplotype frequency to that reported in a larger study conducted by Gonzalez et al. (1999, 2001) were observed. In SAC, HHA appears to be underrepresented (4.3% vs. 10% reported in Caucasians (Gonzalez et al., 2001)) and HHC as overrepresented (55.7% vs. 35% in Caucasians (Gonzalez et al., 2001)) and in SAA, HHF appears underrepresented (15.7% vs. 24% reported in African non-pygmies (Gonzalez et al., 2001)) and HHG*1 overrepresented at 4.3% (2% reported in African non-pygmies (Gonzalez et al., 2001)). Fisher exact test shows significant difference in HHC frequencies (*P* = 0.005) but no significant difference in the HHF, HHG*1 and HHA frequencies between the two studies (*P* > 0.05). The overrepresentation of HHC haplotype frequency in the SAC population in this study could potentially be attributed to differences in Caucasian population ancestry in the two studies. In the larger Gonzalez et al. (2001) study, their Caucasian study group (*n* = 959) is comprised of HIV-1 uninfected individuals from Finland, France and Poland and European American individuals of mixed infection status (i.e. both HIV-positive and HIV-negative individuals) (Gonzalez et al., 2001). Another possible explanation would be the presence of a greater amount of admixture within the Caucasian study group in the Gonzalez et al. (2001) study. These previously defined haplotypes however are located in a relatively small region of the *CCR5* gene (898 bp of the regulatory region of *CCR5*, in addition to presence/absence of CCR5Δ32 in ORF and *CCR2* V64I upstream on the same chromosome). This study has identified putative haplotypes which extend over the entire gene in both directions.

Seven complex putative haplotypes spanning the length of the sequenced region have been identified (Fig. 2B). These haplotypes appear to be extensions of haplotypes previously described within *CCR5* (Gonzalez et al., 1999) (HHA, HHC, HHD, HHE and HHG*2). Haplotypes were named by prefixing the root haplotype name with the population within which it was found. Thus, a distinction can be made when haplotypes with the same root differ in SNP composition between study population groups (e.g. SAA-HHE and SAC-HHE which are both rooted on the HHE haplotype but differ between SAA and SAC individuals bearing the HHE haplotype). Where haplotypes were found to be identical in both study populations, the prefix SAA/C– was used. All the haplotypes described in this study occurred at a frequency greater than 5% and one was found at a frequency of 52.9% (SAA/C–HHC in SAC individuals). Five predominant putative haplotypes were identified in the SAA population whereas only three were identified in the SAC population (Fig. 2B). Only one haplotype appears to be shared by both study populations. This haplotype, SAA/C–HHC, is comprised of three indels and eight SNPs and is the most frequent haplotype in SAC individuals (52.9%) and the least frequent in SAA individuals (8.6%) (Fig. 2B).

Linkage disequilibrium analysis between every two SNP combination in haplotypes identified in this study demonstrated strong linkage disequilibrium between SNPs with a statistical significance greater than 95%.

All HHA, HHF, HHG*2 and HHD haplotypes were found to be associated with further SNPs, forming haplotypes SAA-HAA, SAA-HHF, SAC-HHG*2 and SAA-HHD, respectively (Fig. 2B). The majority of HHC and HHE alleles are associated with further SNPs forming SAA/C–HHC, SAC-HHE and SAA-HHE. SAC-HHE and SAA-HHE are two putative haplotypes rooted on the HHE haplotype but differ at the inclusion of an additional SNP (−4358A/G) in SAC-HHE. The corresponding haplotypes occur at similar frequencies in the two populations (SAC-HHE: 18.6% in SAC and SAA-HHE: 20% in SAA).

All 13 alleles (SAA) classed as HHD can also be classed as SAA-HHD. Hence, a further three polymorphisms −4088T/C; −3894T/C and −3261G/A) can be said to be associated with the HHD haplotype (*D*′ = 1.0, *P* < 0.0005, for all SNP associations within the haplotype). No HHD haplotypes were found in the SAC population. HHF haplotypes appear to be linked to the +2919T/G SNP forming SAA-HHF.

In the SAC population 37/39 alleles classed as haplotype HHC, could also be classed as the HHC extended haplotype, SAA/C–HHC, by far the most predominant haplotype in that population (Fig. 2B). The remaining 2 HHC-bearing alleles occurred in two individuals homozygous for ten of the eleven polymorphism sites comprising SAA/C–HHC and were heterozygous for the +2077T/G SNP. Six out of eight HHC alleles in the SAA population exhibit the SAA/C–HHC polymorphism pattern, one allele lacks the SNP at position +2077 and the other lacks the SNP at −3458.

The HHA haplotype, which comprises WT bases at all SNPs positions used in the Gonzalez et al. (1999) classification system, is present at a frequency of 24.3% and 4.3% in the SAA and SAC populations, respectively (Fig. 2A). Although this may appear to imply that individuals harbouring the HHA haplotype are WT across the entire *CCR5* gene, this is not the case as the HHA haplotype appears to be associated with different SNPs in the extended haplotypes in the different populations (Fig. 2B). In SAA individuals, HHA is associated with SNPs: −1686A/C, −1464A/G; −113G/T and +1752G/A forming SAA-HHA, whereas in SAC HHA alleles demonstrate no association with those SNPs but are instead linked in a haplotype to C/T SNPs at positions −3886; −1060 and +1823, all of which are polymorphisms not detected within the SAA study group. It must be noted, however, that in the SAC population this is a rare haplotype (4.3%) and so has not been shown as one of the predominant haplotypes in Fig. 2B and caution must be taken in assuming this is a true association.

The haplotype HHG can be subdivided into HHG*1 (alleles not containing Δ32 deletion in ORF) and HHG*2 (alleles containing Δ32 deletion in ORF). All HHG*2 haplotypes detected in this study (*n* = 5) were found to contain an additional three SNPs (−5268G/A, −4257A/C and +2919T/G), forming SAA-HHG*2 (Fig. 2B). The two SAC-HHG*1 alleles were identical to SAA-HHG*2 with one individual lacking the +2919T/G polymorphism. In contrast, the three SAA-HHG*1 alleles only had the additional polymorphisms, −5268G/A and −2852A/G, in common with the SAA-HHG*2 haplotype.

### 3.4 Hardy–Weinberg equilibrium

No significant deviations from Hardy–Weinberg equilibrium were noted for any of the indels or SNP loci detected in this study in both the SAA and SAC population groups.

## 4 Discussion

In this study we have characterized polymorphisms (SNPs and indels) and intragenic haplotypes found within *CCR5* for two South African populations, SAA and SAC. This provides a baseline study for the *CCR5* polymorphism and haplotype profiles within these two populations. Previously unreported polymorphisms have been identified and previously defined haplotypes within the *CCR5* gene have been expanded upon.

There exists greater genetic diversity and low levels of linkage disequilibrium within African populations in comparison to European-originating populations (Tishkoff and Verrelli, 2003; Tishkoff et al., 2009). Hence, it is not unexpected to have found a greater number of polymorphisms in SAA in comparison to SAC individuals, as also observed in a recent study reported by Paximadis et al. (2009). Full length sequencing of the *CCR5* gene allows for identification of SNPs which would normally not be detected. Although a number of NI SNPs were identified in our study population, these may have been missed in previous studies which look at a smaller portion of the gene or which use other means of identifying specific polymorphisms. Also, most of the NI polymorphisms identified in this study were detected exclusively in the SAA population. Owing to the sample sizes in this study, detection of a SNP in only one of the two study population groups cannot be used to conclude that that SNP is absent in the other population, but it can be used as an indication of overall prevalence and diversity.

At SNP positions where the major allele (WT) in our study and that of other human reference sequences differed from that of the chimpanzee sequence, the chimpanzee sequence was found to correspond to the minor allele in humans (CTAT/–, AG/– and ACAA/G indels as well as −2852A/G and −113G/T SNPs). In a study characterizing the SNPs in 106 human genes, Cargill et al. (1999) noted that in a significant fraction of cases, the minor chimpanzee allele had become the major human allele and hence the minor human allele was in fact the older allele. This has also been observed in a study reporting variants in the *CCL3* and *CCL3L* genes which code for CCR5 ligands (Paximadis et al., 2009).

The codon 335 mutation resulting in an alanine to valine (A335V) substitution was previously reported by Ansari-Lari et al. (1997) and has been found to be present at a higher frequency in African American populations in comparison to Caucasians (Ansari-Lari et al., 1997; Carrington et al., 1997; Carrington et al., 1999). Although this mutation occurs in the ORF and could be thought to potentially affect protein structure and/or function, in a disease association study, this mutation has been found to have no effect on the rate of progression to AIDS (Carrington et al., 1997). In a study conducted in South African populations, the A335V mutation was detected in African and Coloured populations but not in Caucasians (Petersen et al., 2001). Our study indicates that this mutation is in fact present in the SAC population but as a very rare polymorphism (only 1/35 individuals harboured this allele). In SAA individuals, this mutation was detected at a much higher frequency than that reported elsewhere (Ansari-Lari et al., 1997; Carrington et al., 1997, 1999; Petersen et al., 2001). However, comparison of A335V mutation frequencies in SAA individuals from this study to that of healthy SAA individuals in another South African study (Petersen et al., 2001) indicated no significant difference between observed frequencies (7.1% vs. 1.6%; *P* = 0.099). This and the Petersen et al. (2001) study were conducted in two widely separated geographical regions within South Africa, the Gauteng and Western Cape provinces, respectively.

A novel non-synonymous mutation has been detected within the ORF at codon 86. It is unclear whether this tryptophan to cysteine amino acid substitution will have an impact on chemokine-receptor function. Amino acid alignment of chemokine receptors, CCR5, CCR2B, CCR1, CCR3, CCR4 and CXCR4 (Carrington et al., 1997) shows that this mutation occurs within a highly conserved region (second transmembrane region) between the receptors at a point where all aligned proteins contain a tryptophan residue. This high level of conservation implies that this region is important to the structure or function of the protein and hence indicates that the significance of this novel mutation warrants further study. Both tryptophan and cysteine residues are hydrophobic molecules but cysteine is considerably smaller than tryptophan. Also, tryptophan residues positioned near lipid bilayers, as with residue 86, tend to form hydrogen bonds with the lipid head groups (Schiffer et al., 1992) whereas cysteine residues are likely to form disulphide bonds. Thus, it is possible that this amino acid change may have an impact on the folding of the peptide chain and hence its function as a receptor.

Several SNPs located in the *CCR5* promoter have been previously reported to affect the expression of CCR5. One such polymorphism is the −2459G/A polymorphism located within the downstream promoter (P1). This polymorphism has been linked to differences in *CCR5* expression levels on CD14^+^ monocytes (Salkowitz et al., 2003) and has known association with the rate of progression to AIDS (McDermott et al., 1998). Individuals homozogous for the −2459G allele exhibit lower *CCR5* receptor density in CD14^+^ monocytes (Salkowitz et al., 2003) and have been linked to slower disease progression (McDermott et al., 1998; Clegg et al., 2000; Knudsen et al., 2001). Both WT and mutant alleles have been found to be present at high frequencies in all racial groups, with reported frequencies of 43% and 57% in African and Caucasian populations, respectively (McDermott et al., 1998). In this study, the −2459A allele was detected at a frequency of 42.9% and 40.0% in SAA and SAC population groups, respectively. Fisher exact analysis of the frequencies observed in the two studies has shown no statistical difference between the African population (*P* = 1.0) but there was a statistical difference between the Caucasian populations (*P* = 0.0083). This is not unexpected as the WT alleles, −2459G and −2135T, which are in very strong linkage disequilibrium with each other (Gonzalez et al., 1999; Clegg et al., 2000), form part of the SAA/C–HHC haplotype which was present at a higher than expected frequency in the SAC population. Thus, it follows that the minor/mutant alleles at those positions will also be underrepresented. These two SNPs form part of the HHE, HHF and HHG haplotypes defined by Gonzalez et al. (1999) and the predominant putative haplotypes, SAC-HHE and SAC-HHG*2 described in the SAC population.

Although studies looking at individual polymorphisms on susceptibility to HIV-1 infection and the rate at which individuals progress to AIDS, do provide useful information, it is not always possible to pinpoint the cause of the observed effect of a particular polymorphism when studying the regulatory region of the gene as different combinations of polymorphisms may be in linkage disequilibrium forming haplotypes. Thus, it is important to look at haplotypes and their prevalence across the breadth of a gene.

When examining the effects of *CCR5* haplotypes on HIV-1 disease in different population groups, Gonzalez et al. (1999) observed that haplotype diversity is greatest in African populations. This was also reflected in our study where five major (frequency >5%) haplotypes were observed in SAA individuals, whereas only three were found in SAC individuals.

In this report there are a number of polymorphisms where the minor allele in the SAA population has been shown to be the predominant or ‘major’ allele in the SAC population. This is most evident with the SNPs comprising the SAA/C–HHC haplotype. While SAA/C–HHC, and hence its associated polymorphisms, is by far the most prevalent haplotype detected in the SAC population (52.9%), in the SAA population this haplotype is much less prevalent (8.6%). The most prevalent haplotype in the SAA population is SAA-HHA which is an extension of the HHA haplotype reported to be the ancestral *CCR5* haplotype (Mummidi et al., 2000). This is likely to be due to different evolutionary pressures being exerted on the two populations or a genetic bottleneck where a significant number of members of one the populations was unable to reproduce.

Previous reports have defined haplotypes within the *CCR5* gene (Martin et al., 1998; Gonzalez et al., 1999). Martin et al. (1998) described 10 haplotypes, CCR5P1–P10, comprising 10 SNP positions within the region starting at Exon 1 and ending in Exon 2B of the gene. The nine haplotypes described by Gonzalez et al. (1999) comprise seven SNP positions within a similar region but extending slightly into Intron 2, the presence/absence of *CCR5*Δ*32* in addition to the *CCR2-64I* mutation. In this study we have expanded upon this and have linked previously defined haplotypes to SNPs both upstream and downstream of these regions forming haplotypes which extend over a larger region of the gene with SNPs linked in a haplotype being as much as ∼8.1 kb apart (SNP positions −5248 and +2919 in Hap-Δ32).

A better understanding of the role played by host genes in response to human immunodeficiency virus (HIV-1) exposure will contribute towards a better understanding of the protective immunity to HIV-1 and of the disease process in HIV-1-infected individuals. CCR5 is increasingly being shown to play a critical and central role in HIV-1 infection and to date a number of genetic mutations within the gene have been found to positively or negatively influence an individual's susceptibility and rate of disease progression. Thus, studies such as these which provide valuable new information regarding the genetic diversity within this gene, are important to the further understanding of the impact of CCR5 expression on host susceptibility to HIV-1.

## Figures and Tables

**Table 1:** Frequencies of identified polymorphisms within the SAA and SAC study populations.

Location on gene	SNP position	Base change (wt/mut)	Accession number^a^	*n* (population frequency)^b^	
				SAA^c^	SAC^c^
5′ UTR (2762 bp)	−5268	G/A	rs3136535	3 (0.043)	6 (0.086)
	−5266	G/A	rs6776227	3 (0.043)	0
	−5214	T/C	NI	1 (0.014)	0
	−5080	T/A	rs41429449	4 (0.057)	0
	−5072	C/T	rs35078594	4 (0.057)	0
	−4897	G/A	NI	0	1 (0.014)
	−4808	G/A	NI	2 (0.029)	6 (0.086)
	−4745	C/T	rs3136536	9 (0.129)	0
	−4630	T/C	NI	0	1 (0.014)
	−4358	A/G	rs7637813	2 (0.029)	16 (0.229)
	−4257	A/C	rs41490645	0	6 (0.086)
	−4223	C/T	NI	4 (0.057)	0
	−4088	T/C	rs41499550	14 (0.200)	0
	−3949	A/G	NI	0	1 (0.014)
	−3899	A/C	rs72622924	10 (0.143)	42 (0.600)
	−3894	T/C	rs41395049	14 (0.200)	0
	−3886	C/T	NI	0	3 (0.043)
	−3868	CTAT/–	rs10577983	10 (0.143)	40 (0.557)
	−3833	C/T	NI	1 (0.014)	0
	−3458	G/T	rs2734225	9 (0.129)	39 (0.557)
	−3432	T/C	NI	1 (0.014)	0
	−3261	G/A	rs41475349	14 (0.200)	0
	−2852	A/G	rs2227010	19 (0.271)	25 (0.357)
	−2823	T/A	NI	1 (0.014)	0

Exon 1 (57 bp)	−2733	A/G	rs2856758	3 (0.043)	7 (0.100)

Intron 1 (501 bp)	−2577	T/G	NI	1 (0.014)	0
	−2554	G/T	rs2734648	22 (0.314)	39 (0.557)
	−2459	G/A	rs1799987	30 (0.429)	28 (0.400)
	−2454	G/A	NI	3 (0.043)	1 (0.014)

Exon 2A (235 bp)	−2150	A/G	NI	1 (0.014)	0
	−2135	T/C	rs1799988	30 (0.429)	28 (0.400)
	−2132	C/T	rs41469351	13 (0.186)	0
	−2086	A/G	rs1800023	9 (0.129)	39 (0.557)
	−2048	C/G	rs41355345	0	1 (0.014)

Intron 2 (1903 bp)	−1835	C/T	rs1800024	11 (0.157)	3 (0.043)
	−1686	A/C	rs9282632	17 (0.243)	0
	−1464	A/G	rs3181037	17 (0.243)	0
	−1193	C/T	NI	1 (0.014)	0
	−1130	AG/–	rs3054375	9 (0.129)	39 (0.557)
	−1060	C/T	rs2856762	0	3 (0.043)
	−976	C/T	rs2254089	9 (0.129)	39 (0.557)
	−975	G/A	rs41395249	6 (0.086)	0
	−730	A/T	NI	2 (0.029)	0
	−651	C/T	rs2856764	9 (0.129)	39 (0.557)
	−451	C/T	NI	3 (0.043)	1 (0.014)
	−444	G/A	rs2856765/rs35046662	9 (0.129)	39 (0.557)
	−362	ACAA/G	rs71619644	9 (0.129)	39 (0.557)
	−113	G/T	rs3176763	17 (0.243)	0
	−112	G/A	rs41352147	1 (0.014)	0

Exon 3/ORF (1059 bp)	+225	T/C	rs1800941	1 (0.014)	0
	+258	G/C	NI	0	1 (0.014)
	+319	C/T	Petersen et al. (2001)	1 (0.014)	0
	+554	Δ32	rs333	0	5 (0.071)
	+673	C/T	Petersen et al. (2001)	1 (0.014)	0
	+1004	C/T	rs1800944	5 (0.071)	1 (0.014)

3′ UTR (2651 bp)	+1253	A/G	NI	1 (0.014)	0
	+1752	G/A	rs41495153	18 (0.257)	0
	+1810	G/A	NI	1 (0.014)	0
	+1823	C/T	rs17765882	0	3 (0.043)
	+1843	G/A	rs41418945	9 (0.129)	0
	+1846	G/A	rs41466044	9 (0.129)	0
	+2066	G/A	NI	2 (0.029)	0
	+2077	G/T	rs1800874	9 (0.129)	37 (0.529)
	+2225	T/C	rs41535253	4 (0.057)	0
	+2293	A/G	rs41526948	0	1 (0.014)
	+2381	A/G	NI	1 (0.014)	0
	+2435	T/A	NI	1 (0.014)	0
	+2458	A/C	rs3188094	6 (0.086)	0
	+2676	C/A	rs41442546	0	6 (0.086)
	+2772	G insertion	NI	5 (0.071)	1 (0.014)
	+2838	C/G	rs41512547	3 (0.043)	0
	+2919	T/G	rs746492	28 (0.400)	27 (0.386)
	+3132	T/G	NI	0	1 (0.014)

a Accession numbers of SNPs detected in this study which have been previously reported in the SNP database (dbSNP) or reference to report not in database are listed here; NI indicates newly identified polymorphisms not found in dbSNP.

b Frequency was calculated for both populations using total number of alleles, i.e., *n* = 70.

c Grey shading highlights polymorphisms which were found to be restricted to either the SAA or the SAC population group.
