# Supplementary material for: Genetic variation within the gene encoding the HIV-1 CCR5 coreceptor in two South African populations
Source: Infect Genet Evol. 2010 May;10(4):487–94. doi: 10.1016/j.meegid.2010.02.012 (PMC2877777; doi:10.1016/j.meegid.2010.02.012)
Supplement: Supplementary file 1 [file mmc1.doc]

Table S1

*CCR5* primer sets and experimental conditions for polymerase chain reaction amplification of overlapping regions of gene

| Fragment | Primer sequence | Binding position | Amplicon size (bp^*^) | Annealing temperature |
| --- | --- | --- | --- | --- |
| Promoter 2 | f: 5′ AAG AAG CCG CCT ACA GAA TGG 3′ | -5523 to -5503 | 2412 | 65°C  (first 10 cycles)  60°C  (last 20 cycles) |
|  | r: 5′ CCC TCA GCT TTC TCG TCT GG 3′ | -3131 to -3111 |  |  |
| Promoter 1 | f: 5′ CCA AGC ACC AGC AAT TAG C 3′ | -3384 to -3366 | 2189 | 60°C |
|  | r: 5′ TGC CAC CAC AGA TGA ATG TC 3′ | -1215 to -1196 |  |  |
| Intron 2 | f: 5′ GTA ACC TCT CAG CTG CTT G 3′ | -1910 to -1892 | 2036 | 55°C |
|  | r: 5′ GAT GAA CAC CAG TGA GTA GAG C 3′ | +105 to +126 |  |  |
| ORF | f: 5′ GCA CCA TGC TTG ACC CAG TT 3′ | -340 to -321 | 2381 | 60°C |
|  | r: 5′ CAT ATG CTG CAC GAA TAC CTC 3′ | +2020 to +2041 |  |  |
| 3' UTR | f: 5′ GAG ATC CTG GTT GGT GTT GC 3′ | +1844 to +1863 | 1866 | 60°C |
|  | r: 5′ GTA AGT GAC CAG GCC ATG AC 3′ | +3689 to +3709 |  |  |

^*^bp, base pair

Table S2

Flanking sequences of all SNPs detected in this study

|  | SNP Position | Base change (wt/mut) | 5' flanking sequence | 3' flanking sequence |
| --- | --- | --- | --- | --- |
| 5' UTR  (2762 bp) | -5268 | G/A | GTC ACC CAG GTT GGA | TGC AAT GGC CCA ATC |
|  | -5266 | G/A | CAC CCA GGT TGG ART | CAA TGG CCC AAT CTT |
|  | -5214 | T/C | CCT GGG TTC AAG AGA | TCT CCC ACT TCA GCC |
|  | -5080 | T/A | CTC CTG ACC TCA AGT | ATC CAC TCG CCT TGG |
|  | -5072 | C/T | CCT CAA GTA TCC ACT | GCC TTG GCT TCC CAA |
|  | -4897 | G/A | AAC AGC TGC TGT ATG | CAG GGT TTC TGC TCA |
|  | -4808 | G/A | TGT TGA CGA TGC TCT | AAA ATA TGG TCC AGA |
|  | -4745 | C/T | CTT GGA ACA TAG GTG | AGT GAC TAG ACA TGG |
|  | -4630 | T/C | TGA TCA AAA GTT CAT | TCC TAT GGG GTG TCC |
|  | -4358 | A/G | CAA AAG TGG AGT AAC | CAC ACT GCA AAG CTG |
|  | -4257 | A/C | GCC ATA GAA TCA TGT | GTA TTT AGG GTG GAA |
|  | -4223 | C/T | GCC CCA GGT CTA GCA | GTC ATT TAA CAG ATG |
|  | -4088 | T/C | CTC TGG GGG TGA GTA | GTC TTC ACA TCC TAA |
|  | -3949 | A/G | TCCGGGGGTTCTGC | CAAGTGGATTACCAG |
|  | -3899 | A/C | CTT TCG AAA AAC CAA | GTT GYA TTT ATG CTA |
|  | -3894 | T/C | CGA AAA ACC AAM TTG | ATT TAT GCT ATC TAT |
|  | -3886 | C/T | AAM GTT GYA TTT ATG | TAT (CTAT) TTT CTA TA |
|  | -3868 | CTAT/- | TTG YAT TTA TGC TAT | TTT CTA TAA AAT TTT |
|  | -3833 | C/T | ACC TAT TTT TGA ACT | TTT CAA AAG CAC ACT |
|  | -3458 | G/T | CTA TAT GGG GCG GGG | TGG GGG TGT CTT GAT |
|  | -3432 | T/C | TTG ATC GCT GGG CTA | TTC TAT ACT GTT CTG |
|  | -3261 | G/A | ACT CTC TCT GAC AAA | GAC TGC TCA AAG AGT |
|  | -2852 | A/G | CAG ATG TCA CCA ACC | CCA AGA GAG CTT GAT |
|  | -2823 | T/A | TAT GAC TGT ATA TAG | ATA GTC ATA AAG AAC |
| Exon 1  (57 bp) | -2733 | A/G | ATA TCT GGA GTG AAG | ATC CTG CCA CCT ATG |
| Intron 1  (501bp) | -2577 | T/G | CCG TGA GCC CAT AGT | AAA ACT CTT TAG ACA |
|  | -2554 | G/T | TTT AGA CAA CAG GTT | TTT CCG TTT ACA GAG |
|  | -2459 | G/A | GTG GAG AAA AAG GGG | CAC ARG GTT AAT GTG |
|  | -2454 | G/A | GAA AAA GGG GRC ACA | GGT TAA TGT GAA GTC |
| Exon 2A  (235bp) | -2150 | A/G | TAA TCC AGT GAG AAA | GCC CGT AAA TAA ACT |
|  | -2135 | T/C | AGC CCG TAA ATA AAC | TTY AGA CCA GAG ATC |
|  | -2132 | C/T | CCG TAA ATA AAC YTT | AGA CCA GAG ATC TAT |
|  | -2086 | A/G | AAG CTC AAC TTA AAA | GAA GAA CTG TTC TCT |
|  | -2048 | C/G | TTC GCC TTC AAT ACA | TTA ATG ATT TAA CTC |
| Intron 2  (1903bp) | -1835 | C/T | GAA ACC CAT AGA AGA | ATT TGG CAA ACA CCA |
|  | -1686 | A/C | TTT AAA GGG AGC AAT | GTA TTT TAA TAA CTA |
|  | -1464 | A/G | ATG GTC AAA ATT AAT | TTA AAT TAC AAA CGC |
|  | -1193 | C/T | ATC TGT GGT GGC AGA | GAA ACA TTT TTT ATT |
|  | -1130 | AG/- | TAA TTG TGG CAA CTC | AAA CTA CAA ACA CAA |
|  | -1060 | C/T | TCA TCT ATG ACC TTC | CTG GGA CTT GGG CAC |
|  | -976 | C/T | TTT TAA TTC TCT TTT | RAG GAC TGA GAG GGA |
|  | -975 | G/A | TTT AAT TCT CTT TTY | AGG ACT GAG AGG GAG |
|  | -730 | A/T | CCC TCG AGG CCT CTT | ATT ATT ACT GGC TTG |
|  | -651 | C/T | AGA CTG AGT TGC AGC | GGG CAT GGT GGC TCA |
|  | -451 | C/T | AGG ATT GCT TGA GCC | GGG ATG RTC CAG GCT |
|  | -444 | G/A | CTT GAG CCY GGG ATG | TCC AGG CTG CAG TGA |
|  | -362 | ACAA/G | CTC ACA ACA ACA ACA | CAA CAA AAA GGC TGA |
|  | -113 | G/T | GTA GAC ATC TAT GTA | RCA ATT AAA AAC CTA |
|  | -112 | G/A | TAG ACA TCT ATG TAK | CAA TTA AAA ACC TAT |
| Exon 3/ORF  (1059bp) | +225 | T/C | CAA CCT GGC CAT CTC | GAC CTG TTT TTC CTT |
|  | +258 | G/C | TAC TGT CCC CTT CTG | GCT CAC TAT GCT GCC |
|  | +319 | C/T | CAA CTC TTG ACA GGG | TCT ATT TTA TAG GCT |
|  | +554 | ∆32 | CTC ATT TTC CAT ACA | TTA AAG ATA GTC ATC |
|  | +673 | C/T | ACT CTG CTT CGG TGT | GAA ATG AGA AGA AGA |
|  | +1004 | C/T | AGG CTC CCG AGC GAG | AAG CTC AGT TTA CAC |
| 3' UTR  (2651bp) | +1253 | A/G | CC CAT CAA TTA TAG | AAG CCA AAT CAA AAT |
|  | +1752 | G/A | CTG TAG AAG GAG ACA | AGC TGG TTG GGA AGA |
|  | +1810 | G/A | ATG AAG AAC CTT GAC | GCA TTG CTC CGT CTA |
|  | +1823 | C/T | ACR GCA TTG CTC CGT | TAA GTC ATG AGC TGA |
|  | +1843 | G/A | TCA TGA GCT GAG CAG | GAR ATC CTG GTT GGT |
|  | +1846 | G/A | TGA GCT GAG CAG RGA | ATC CTG GTT GGT GTT |
|  | +2066 | G/A | AGT CAG CAG AAC TGG | GTG GAT TTG GKT TGG |
|  | +2077 | G/T | CTG GRG TGG ATT TGG | TTG GAA GTG AGG GTC |
|  | +2225 | T/C | AAA GGA TGG GTC TGG | TTG CAG AGC TTG AAC |
|  | +2293 | A/G | TGC TTC TGA CTT CAT | GAT TTC CTT CCC ATC |
|  | +2381 | A/G | ATG AGG TCT AGG AAC | TAC TTC AGC TCA CAC |
|  | +2435 | T/A | TAC CTA GTA GTC ATT | CAT GGG TTG TTG GGA |
|  | +2458 | A/C | TGT TGG GAG GAT TCT | TGA GGC AAC CAC AGG |
|  | +2676 | C/A | AAA GGG GGG AAG GGA | ATA TTC ATT TGG AAA |
|  | +2772 | G insertion | TGG GGG TGG GGG GGG | CGC CTT AGG TAC TTA |
|  | +2838 | C/G | GAA AAA ATC GTC TCT | CCT CCC TTT GAA ATG |
|  | +2919 | T/G | GTT TTT TTC TGT TCT | TCT CAT ATG ATT GTG |
|  | +3132 | T/G | CAA CGA AGG GAA ATG | CTT TCC TTT TGC TCT |
